# Supplementary material for: Sodium Accumulation in Infected Cells and Ion Transporters Mistargeting in Nodules of Medicago truncatula: Two Ugly Items That Hinder Coping with Salt Stress Effects
Source: Int J Mol Sci. 2022 Sep 13;23(18):10618. doi: 10.3390/ijms231810618 (PMC9505113; doi:10.3390/ijms231810618)
Supplement: Supplementary file 1 [file ijms-23-10618-s001.zip › ijms-1803167-supplementary.pdf]

Supplementary material figure and table legends.

Table S1. Gene identifiers or locus names and accession numbers of *M. truncatula* (A) and *S.meliloti* (B)genes used in this study.

Table S2 Genes enriched in infected (A) and non-infected cells(B) of the fixation zone (extraction from Supplementary data of Limpens et al., 2013**).**

Table S3 Expression of *M. truncatula* genes encoding proteins of the inward transport of potassium, cation/sodium exchangers and putative regulators in the nodule developmental zones. Data (deseq-normalized RNA-seq reads) were obtained from Symbimics database. FI: Meristem; FIId: Distal infection zone; FIIp: Proximal infection zone; IZ: Interzone II-III; ZIII: nitrogen-fixing zone, mature zone.

Table S4. Expression of potassium transporters/exchangers genes of *Sinorhizobium meliloti* in root nodule developmental zones. Data (deseq-normalized RNA-seq reads) were obtained from Symbimics data base. FI: Meristem; FIId: Distal infection zone; FIIp: Proximal infection zone; IZ: Interzone II-III; ZIII: nitrogen-fixing zone.

Table S5. Relative distribution of elements in *M truncatula* nodules of plants treated with 0 or 100 mM NaCl for 2 weeks.

Table S6 List of primers used in this research.

Figure S1 (A,B). Western blot analysis of MtNHX6 in *M. truncatula* roots with anti-NHX6 antibody. The band around 58 kD is marked by arrowhead (A); location of the peptide in the protein sequence of the exchanger highlighted by yellow (B).

Table S1. *M. truncatula* (A) and ***S. meliloti*** (B) genes used in this study.

A

**Protein**	**Phytozome** Gene ID or *Locus* name, genome v.Mt4.0	**Gene ID§,** genome v.Mt5.0	**NCBI Protein seq**	**Symbimics Accession**	**Homologe *A. thaliana* to Uniprot**
Transporters					
**MtKT2**	Medtr3g094090	3g0128831	XP_013461519.1	Mt0007_10301	O22881
**MtKT3**	Medtr8g099090	8g0387661	XP_013447138.1	Mt0009_10672	Q9FE38
**MtHAK5**	Medtr4g099260	4g0055471	XP_013457577.1	Mt0001_01559	Q9M7K4
**MtHAK6**	Medtr5g034500	5g0413721	XP_003613249.1	Mt0012_10601	Q8W4I4
**MtHAK7**	Medtr2g008820	2g0279131	XP_003593196.2	Mt0023_10359	Q9FY75
**MtHAK8**	Medtr6g007697	6g0451081	XP_013450832.1	Mt0025_00124	Q9M7J9
**MtHKT6**	Medtr6g092840	6g0488681	XP_013453240.1	Mt0024_10466	Q84TI7
Channels					
**MtAKT1**	Medtr4g113530	4g0063141	XP_003609240.1	Mt0001_00981	Q38998
**MtAKT2/3**	Medtr2g006870	2g0277651	XP_003593018.1	Mt0023_00412	Q38898
**MtKAT1**	Medtr8g446430	8g0356081	XP_013445265.1	Mt0015_00521	Q39128
**MtKAT3**	Medtr3g108320	3g0138671	XP_003603500.1	Mt0007_10989	P92960
**MtSKOR/GORK**	Medtr5g077770	5g0434371	XP_003616247.2	Mt0063_10132	Q94A76
Exchangers					
**MtCHX18**	Medtr5g009770	5g0397041	XP_024638960.1	Mt0005_00234	Q9FFR9
**MtNHX1**	Medtr1g081900	1g0191231	KEH43058.1	Mt0004_00150	Q68KI4
**MtNHX2**	Medtr7g114250	7g0273581	XP_024626893.2	Mt0036_10161	Q56XP4
**MtNHX3**	Medtr4g118770	4g0066681	XP_024638068.1	Mt0001_00738	Q84WG1
**MtNHX4***	Medtr3g055600	3g0100591	XP_003600216.2	Mt0013_00709	Q8S397
**MtNHX6**	Medtr2g028230	2g0291241	XP_024632389.1	Mt0018_10150	Q8RWU6
**MtNHX7**	Medtr2g038400	2g0298911	AES65365.2	Mt0055_10095	Q9LKW9
**MtNHX7-like**	LOC120577421	-	XP_039684716.1	-	Q9LKW9
**MtNHX8**	LOC120577420	8g0375421	XP_039684715.1	Mt0020_00485	Q3YL57

*** This gene is annotated as MtNHX3 in Symbimics**

§ IDs obtained from hhttps://medicago.toulouse.inra.fr/MtrunA17r5.0-ANR. The prefix 'MtrunA17_Chr' was omitted for simplicity; i.e. ID of MtKT2 is: MtrunA17_Chr3g0128831

B

	NCBI Protein seq	Symbimics	Uniprot *R. meliloti*
**Putative potassium transport system protein kup1**	AEH77766.1	SMc00873	Q92RN0
**Putative potassium transport system protein kup 2**	WP_010967951.1	SMa1798	Q92Y93
**Potassium-transporting ATPase KdpC subunit**	WP_010968196.1	RA1253, SMa2329	Q52980
**Putative subunit A/B (pH adaptation potassium efflux system protein A/B, Pha system subunit A/B)**	WP_010970284.1	SMc03179	Q52978
**Na(+)/H(+) antiporter NhaA (Sodium/proton antiporter NhaA)**	MQW43809.1	SMa1913	Q92Y37

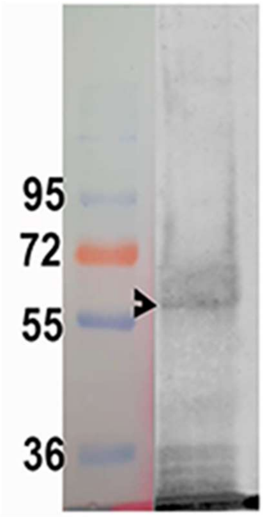

NHX6 - Medtr2g028230.1- MTR_2g028230- AES64654.2

```
MGSENEISPADVHKAPPGKEQQAAGVGILLQIMMLVLSFVLGHVL
RRKKIYIIPEASASLLIGLIVGILANISDTETNIRAWFNFHEEFF
FLFLLPPIIFQSGFSLAPKPFFSNFGAIVTFAIFGTFLASFVTGA
LVYLGGLVFLMYRLPFVECMMFGALISATDPVTVLSIFQELGTDV
NLYALVFGESVLNDAMAISLYRTMSVVKNNPSGQNFFMVVVRFLE
TFVGSLSAGLDIDNLQNLESCLFVLFPYFSYMLAEGLGLSGIVSI
LFTGIVMKHYAYSNLSQSSQRFVSAFFELISSLAETFIFIYMGFD
IAMEQHSWSHVGFIFFSVIFIVIARAANVFSCAYLVNLVRPAHRK
IPPKYQKALWYSGLRGAMAFALALQSVHDLPEGHGQTIFTATTAI
VVLTVLLIGGSTGTMLEALDVIGGDTHSDSSLASVGPITDYGGNN
GYIAPSYEEESSSSGNKIKMKLKEFHKSTASFTALDRNYLTPFFT
SHNGDEDEEAEPFTSARSFTSSRSGFHGQSPYASP
```

Figure S1 (A,B). Western blot analysis of MtNHX6 in *M. truncatula* roots with anti-NHX6 antibody. The band around 58 kD is marked by arrowhead (A); location of the peptide in the protein sequence of the exchanger highlighted by yellow (B).

Table S2. Genes enriched in infected (A) and non-infected cells (B) of the fixation zone (extraction from Supplementary data of Limpens et al., 2013)

. A

Gene	number	Infected Cells P value	Uninfected Cells P value
Ca2+/H+ antiporter VCX1 and related proteins	mtr.37432.1.s1_at	3,27	0,928
Ca2+/H+ antiporter VCX1 and related proteins	mtr.41148.1.s1_at	9,41	1,776
Mn2+ and Fe2+ transporters of the NRAMP family	mtr.17288.1.s1_at	3,45	0,599
Fe2+/Zn2+ regulated transporter	mtr.37432.1.s1	7,77	1,346
Vacuolar cation/proton exchanger 3	mtr.41148.1.s1_at	2,70	0,594
K(+)/H(+) antiporter 1	mtr.48853.1.s1_at	6,48	1,062
Potassium transporter 6	Mtr.11919.1.S1_at	9,66	0,983
Sodium/hydrogen exchanger 1	mtr.21664.1.s1_at	3,42	0,769

B

Gene	Number	Infected Cells P value	Uninfected Cells P value
Copper transporter 1<br>metal transporter family protein<br>K(+)/H(+) antiporter 1<br>Zinc transporter 5<br>ATPase, Cu++ transporting, heavy-metal-associated<br>heavy-metal-associated domain-containing protein / copper chaperone (CCH)-related<br>heavy-metal-associated<br>Magnesium transporter MRS2 homolog, mitochondrial<br>MRS2-like protein<br>heavy-metal-associated<br>heavy-metal-associated<br>heavy-metal-associated<br>Zinc transporter 11<br>Zinc transporter 1<br>heavy-metal-associated<br>heavy-metal-associated<br>heavy-metal-associated<br>heavy-metal-associated<br>MATE efflux family protein	mtr.17748.1<br>mtr.1998.1<br>mtr.20201<br>mtr.38036.1.<br>mtr.47879.1.s1_<br>Mtr.13235.1.S1_<br>Mtr.15870.1.S1_<br>Mtr.12042.1.S1_<br>Mtr.27738.1.S1_<br>Mtr.30784.1.S1_at<br>Mtr.34165.1.S1_at<br>Mtr.26319.1.S1_at<br>Mtr.37582.1.S1_at<br>Mtr.40995.1.S1_at<br>Mtr.43988.1.S1_at<br>Mtr.43347.1.S1_s_at<br>Mtr.43348.1.S1_at<br>Mtr.49487.1.S1_at<br>mtr.8402.1.s1_at	0,589<br>1,900<br>0,680<br>0,422<br>0,343<br>0,589<br>1,900<br>0,680<br>0,422<br>1,078<br>1,444<br>0,398<br>1,266<br>0,343<br>0,673<br>0,609<br>4,640<br>1,483<br>0,604<br>0,411	6,367<br>4,082<br>18,287<br>5,520<br>12,529<br>6,367<br>4,082<br>18,287<br>4,874<br>12,082<br>4,870<br>7,183<br>12,112<br>13,751<br>3,559<br>18,454<br>65,468<br>20,315<br>11,246<br>9,953

Table S3. Expression of potassium transporters/exchangers genes of *Sinorhizobium meliloti* in root nodule developmental zones. Data (deseq-normalized RNA-seq reads) were obtained from Symbimics data base. FI: Meristem; FIId: Distal infection zone; FIIp: Proximal infection zone; IZ: Interzone II-III; ZIII: nitrogen-fixing zone.

**Nodule zones** / **Gene name,** ID	**FI**	**FIId**	**FIIp**	**IZ**	**ZIII**
**Putative potassium transport system protein kup 1** (SMc00873)	169.9±34.5	146.6±19.9	194.6±61.7	313.8±32.6	863.6±54.1
**Putative potassium transport system protein kup 2** (SMa1798)	131.1±91.1	151.8±30.7	70.3±24.0	221.7±38.1	1855.6±165.7
**Potassium-transporting ATPase KdpC subunit** (RA1253, SMa2329)	0	1.2±0.6	1.,3±0.8	1.5±0.4	14.0±2.5
**Putative subunit A/B (pH adaptation potassium efflux system protein A/B, Pha system subunit A/B)** (SMc03179)	168.1±34.2	207.1±26.0	172.8±43.6	168.8±2.0	135.4±10.7
**Na(+)/H(+) antiporter NhaA (Sodium/proton antiporter NhaA)** (SMa1913)	264.1±39.1	427.1±73.1	253.4±67.5	350.2±35.8	901.9±50.6

Table S4. Expression of *M. truncatula* genes encoding proteins of the inward transport of potassium, cation/sodium exchangers and putative regulators in the nodule developmental zones. Data (deseq-normalized RNA-seq reads) were obtained from Symbimics database. FI: Meristem; FIId: Distal infection zone; FIIp: Proximal infection zone; IZ: Interzone II-III; ZIII: nitrogen-fixing zone, mature zone.

**Nodule zones** / **Gene name,** ID or locus name	**FI**	**FIId**	**FIIp**	**IZ**	**ZIII**
Transporters					
**MtKT2**, Medtr3g094090	230.6±5.2	60.2±3.4	23.5±7.4	22.7±6.2	12.9±3.8
**MtKT3**, Medtr8g09 9090	83.1±5.3	75.1±7	82.8±6.9	76.3±9.6	68.0±5.8
**MtHAK5,** Medtr4g099260	15.3±0.2	2.9±0.7	10.7±5.4	4.3±1.9	0
**MtHAK6**, Medtr5g034500	114.7±5.4	270.2±11.9	572.2±37.5	1737.2±216.1	3139.4±211.8
**MtHAK7**, Medtr2g008820	68.3±2.2	76.5±1.7	101.6±13.2	92.9±13.2	135.8±22.5
**MtHAK8**, Medtr6g007697	183.8±25.3	63.7±9.4	58.6±46.4	2.4±0.8	7.9±4.3
**MtHKT6**, Medtr6g092840	2.5±0.5	1.4±0.4	0.2±0.2	0	0
Channels					
**MtAKT1**, Medtr4g113530	37.2±5.5	3.2±0.3	2.6±2	10.2±2	8±1.3
**MtAKT2/3**, Medtr2g006870	1.9±0.4	1.7±1.1	1.2±0.7	0	0.5±0.5
**MtKAT1**, Medtr8g446430	4.0±0.6	3.9±1.8	2.5±1.4	1.0±0.5	0
**MtKAT3**, Medtr3g108320	8.0±0.3	3.4±1	0.2±0.2	1.2±0.6	5.1±4.7
**MtSKOR/GORK**, Medtr5g077770	11.9±0.4	8.2±2.3	8.8±6.4	43.6±7.2	24.6±8
*Exchangers**					

**MtCHX18**, Medtr5g009770	61.9±4.7	58.9±10.5	88±17.5	135.6±15.2	116.8±10.8
**MtNHX1**, Medtr1g081900	126.4±7.2	140.5±24.8	190.5±28.9	149.7±13.3	287.9±30
**MtNHX2**, Medtr7g114250	1.7±1.5	0.7±0.7	0.2±0.2	0	0
**MtNHX3** Medtr3g055600	0	0.2±0.2	0	0	0
**MtNHX4**, Medtr4g118770	6.7±1.6	5.9±1.8	0.8±0.8	0	0.3±0.3
**MtNHX6**, Medtr2g028230	69.9±0.7	141.9±17.8	159.3±22.3	139.2±11.2	153.6±6.3
**MtNHX7**, Medtr2g038400	0.5±0.3	0.5±0.2	0.5±0.5	0	0
**MtNHX8**, LOC120577420	68.6±2.7	53±4.6	26.9±11.8	44.0±2.4	62.4±9.4
Putative regulators					
**MtSOS3**, Medtr3g091440	47,2±1	173,7±6	202,7±28	500±26	300±13
**MtbHLH121**, Medtr1g032600	33.5±7.2	44.4±5.6	36.7±9	29.5±4.6	48.4±7.2
**MtbHLH122**, Medtr4g065870	137.2±22.3	150.4±11.9	144.3±8.7	134.9±18.4	149.9±25.0

*, Note that *MtNH7-like* are not available in Symbimics database

Table S5. Relative distribution of elements in *M truncatula* nodules of plants treated with 0 or 100 mM NaCl for 2 weeks.

Nodules of control (0 M NaCl) and treaded (0,1 M NaCl, 15 days) M.truncatula plants														
Nodule organelles	P		Mo		Mg		Cl		Ca		Fe		S	
	Control	NaCl	Control	NaCl	Control	NaCl	Control	NaCl	Control	NaCl	Control	NaCl	Control	NaCl
**Non-infected cells**														
Vacuole	22,6±11	4,1±2,5	11,8±7,8	1,9±1,8	6,3±3,6	17,5±9,5	*7,0±2,9	25,7±4,4	*10,0±6,2	1,9±0,8	2,7±1,8	3,6±2,3	11,7±4,5	15,6±1,8
Cytoplasm	2,5±1,2	3,6±0,87	1,28±0,87	2,65±1,0	2,9±1,34	4,3±2,2	*7,88±1,7	15,8±2,8	3,6±0,9	3,95±1,76	2,5±0,6	4,1±1,7	-	-
**Infected cells**														
Vacuole	2,9±0,7	10,0±5,5	3,6±2	11,9±8,4	8,3±4,5	10,9±6,5	15,25±4,9	10,7±5,0	3,14±1,2	2,7±1,3	3,3±1,3	4,6±1,9	*24,1±3,4	4,9±3,1
Cytoplasm	3,9±1,8	4,0±1,0	5,3±0,7	5,0±1,22	4±1,65	4,9±0,9	*6,8±0,7	13,5±1,6	5,1±1,4	6,7±1,4	*4,8±0,75	8,7±0,9	-	-
Bacteroid	22,5±2,7	22,8±11,3	9,1±1,4	16,6±5,8	6,0±3,0	9,2±5,8	10,6±1,9	13,3±6,4	9,9±4,3	7,8±3,8	4,8±2,3	5,8±3,3	4,0±2,4	8,0±3,8

Table S6. List of primers used in this research.

Cloning primers

MtNHX7

ORF:
R:TAAGTTTAAGAGTCTGAAGTGTG
L:TTGGGATCACAAGTAACTCGGA

Promoter:
R:AAGTTTAAGAGTCTGAAGTGTG
L:TGGGATCACAAGTAACTCGGA

q-PCR primers

MtNHX7 (Medtr2g038400)

F: GATCAAACCCTGCCGAGTAG

R: ACGGGAAAGTCAGCTTCAAA

MtNHX8 **LOC120577420**

F: TGCAGTTATCTTCTTTGGTCTCAG

R: ATCTTTCCCAGCCGATGAT

***MtNHX7-like*: LOC120577421**

MtMtc27 (Medtr TC106535)

F: CGAACGAGGTCTTCAGCATGA

R: ACGAGATCGAGATCGCCATT

MtGAPDH (Medtr4g103920)

F: GTTTTTACCGACAAGGACAAAGCT

R: ACAAACATGGGAGCATCCTTACTAG

MtAKT1 (Medtr4g113530)

F: ACGCCGTGGTTCCAACAT

R: CATTATGCGTTACGGAGGAGACA

MtSKOR/GORK (Medtr5g077770)

F: GCGTCGCTGGAAAATCCTAT

R: TCCACACACCATTCAAGAGCTAA

MtHAK6 (Medtr5g034500)

F:GGTGGAGTTTTGCTGTCCAT

R:CCAGAACAGGCCATCTCAAT

F:

AAGAGGTGATGATTCAGCTATTGA

R: TTCTTCACCACTTTCACCTCAA

MtNHX6 (Medtr2g028230)

F: GCTTGTAAAGAACGGGGTGA

R: GGCTTCAGTTGGTCCCATTA

MtNHX1 (Medtr1g081900)

F: ACGCATGAATGAATCGTCAA

R: CCACAAACAGCATGACAACC

MtHAK7 (Medtr2g008820)

F:ACGAGGGAAACAACCAAGTG

R:CGTGCCTGCTATTGGGTTAT

MtCHX18 (Medtr5g009770)

F: AGAGCCGCAGTCATCATTCT

R: CAAGAGCTTCACGGTCATCA

SMc00128

ACGAGANCGAGATCGCCATT

CGAACGAGGTCTTCAGCATGA
